# Universally Grasping Objects with Granular—Tendon Finger: Principle and Design

**DOI:** 10.3390/mi14071471

**Published:** 2023-07-21

**Authors:** Van Pho Nguyen, Sunil Bohra Dhyan, Boon Siew Han, Wai Tuck Chow

**Affiliations:** 1 School of Mechanical and Aerospace Engineering, Nanyang Technological University, Singapore 639798, Singapore; sunilbohra.dhyan@ntu.edu.sg; 2 Schaeffler Hub for Advanced Research at NTU, Singapore 637460, Singapore

**Keywords:** universal grasp, soft gripper, hybrid gripper, soft robot hand, hybrid robot hand, granular gripper, tendon gripper

## Abstract

Nowadays, achieving the stable grasping of objects in robotics requires an increased emphasis on soft interactions. This research introduces a novel gripper design to achieve a more universal object grasping. The key feature of this gripper design was a hybrid mechanism that leveraged the soft structure provided by multiple granular pouches attached to the finger skeletons. To evaluate the performance of the gripper, a series of experiments were conducted using fifteen distinct types of objects, including cylinders, U-shaped brackets, M3 bolts, tape, pyramids, big pyramids, oranges, cakes, coffee sachets, spheres, drink sachets, shelves, pulley gears, aluminium profiles, and flat brackets. Our experimental results demonstrated that our gripper design achieved high success rates in gripping objects weighing less than 210 g. One notable advantage of the granular-tendon gripper was its ability to generate soft interactions during the grasping process while having a skeleton support to provide strength. This characteristic enabled the gripper to adapt effectively to various objects, regardless of their shape and material properties. Consequently, this work presented a promising solution for manipulating a wide range of objects with both stability and soft interaction capabilities, regardless of their individual characteristics.

## 1. Introduction

Nowadays, soft robotics grippers have been successfully applied to numerous objects due to the soft interactions generated by the robot body. Some of the designs for such grippers were inspired by living organisms [1,2,3]. For instance, researchers proposed soft tree-frog-like pads [4,5,6,7,8,9,10] and gecko-like pads [11,12,13,14,15,16] capable of handling objects in wet and dry conditions through gentle squeezing. Other soft hands [16,17,18,19,20] illustrated their advantages in grasping soft objects such as fruit, food, harvest, etc. These grippers exhibited an elevated level of softness and majorly focused on handling soft objects. To tackle the challenge of handling heavier loads, the authors of [21,22,23,24] designed stronger soft grippers inspired by the arms of an octopus or suction cups. The development of fin ray gripper [25,26,27] achieved better performance in manipulating large objects with a strong force generated from the bodies. While previous works on soft robotic grippers made significant contributions across various fields, two fundamental problems persist: controlling gripper behaviour with infinite degrees of freedom (DoFs) and ensuring stable operation. These limitations significantly impede the soft gripper’s ability in dealing with heavy loads.

The conventional grippers discussed in [28,29,30,31] and caging grippers mentioned in [32,33,34,35,36,37,38,39,40,41] featured fingers or claws composed of rigid phalanges and hard joints. As a result, these grippers could stably hold objects due to the high stiffness of the rigid fingers. This characteristic enabled them to squeeze the objects more strongly and reduce the instability in picking up heavy objects. In addition, controlling these was relatively simpler compared to controlling soft grippers, due to the low DoFs (degrees of freedom) of the robot body. However, the main drawbacks of traditional grippers are that, firstly, the rigid bodies and fingers are not friendly in interacting with the objects, especially delicate or fragile ones, which could cause unexpected outcomes. Additionally, the low DoFs (degrees of freedom) of the robot bodies and fingers restrict the flexibility of the robot hand to adapt to various objects, especially with complex shapes universally.

In recent times, hybrid grippers have emerged as a design approach to inherit the soft and rigid properties of the previously mentioned soft and traditional grippers. The authors of [42] developed a hybrid gripper with two rigid fingers actuated by the shape memory alloy (SMA) springs. Li [43] proposed a gripper in which each finger has a soft–rigid actuator for flexibly adapting multiple objects. This was a combination of the pneunet structure and the heating elements. Tomasz [44] utilized a hybrid biomimetic gripper with five fingers for gripping elastic objects. Furthermore, [45] introduced an origami actuator implanted into the rigid fingers to enable the handling of heavy objects through a hybrid structure. Our previous works [46,47] presented soft hybrid grippers capable of universally grasping various objects, whether individually or in groups. Each finger of this gripper comprised an elastic cord supported by rigid curves. By integrating both soft and rigid structures, these hybrid grippers were advantageous in gripping objects with flexibility and stability. However, while the works majorly focused on the actuator mechanisms, the flexibility of the contact interfaces between the fingers and the objects was not extensively addressed.

### Research Concept

**Figure 1:**
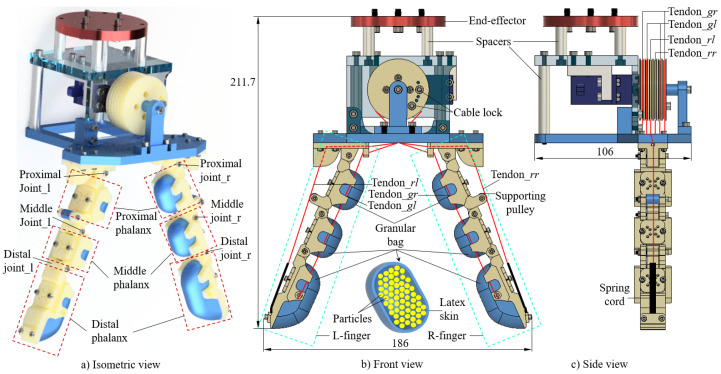
Three-dimensional design of the granular-tendon gripper: (**a**) isometric view, (**b**) front view, and (**c**) side view. The red-dashed line box and the green-dashed line box show the structure of the finger at each phalanx and at the entire finger. Moreover, the red solid lines visualize the tendon trajectories. The group of finger joints on the left finger (L-finger) include proximal joint ${}_{l}$, middle joint ${}_{l}$, and distal joint ${}_{l}$, whereas those on the right finger (R-finger) include the proximal joint ${}_{r}$, middle joint ${}_{r}$, and distal joint ${}_{r}$. The tendon ${}_{gl}$ and tendon ${}_{gr}$ are the respective grasping tendons on the L- and R-finger, whereas tendon ${}_{rl}$ and tendon ${}_{rr}$, respectively, are the releasing tendon on the L- and R-finger.

By integrating granular particles into the robotic hand, the flexibility at the contact interface of the gripper can be enhanced. As illustrated in [48,49,50,51], it moved inside the jamming bead when the finger contacts the objects. With this mechanism, the stiffness of the finger could be varied, and the shape of the fingers could adapt easily to the objects. However, existing research on granular-particle grippers focused on designs with one bulky bag of jamming beads. In contrast, our study presents a novel design of multiple small pouches of granular particles integrated into cable-driven skeleton fingers. This granular-particle gripper allows for the universal manipulation of various objects with high stability and soft–flexible interactions. We designed a robot gripper with two symmetrical hybrid fingers driven by the tendon system. Each finger incorporated three granular bags attached to the phalanx skeleton, which resulted in a granular-tendon gripper (see Figure 1). The gripper was designed and experiments were conducted to evaluate its performance in grasping various objects in each trial with fifteen types of objects: cylinder, U-shaped bracket, M3 bolt, tape, pyramid, big pyramid, orange, cake, coffee sachet, sphere, drink sachet, shelf, pulley gear, aluminium profile, and flat bracket. The experimental results demonstrated that the gripper could bear heavy loads with the help of the rigid skeleton structure driven by the cable system. Additionally, the granular bags generated soft interactions at the surface contacting the objects. Thus, the gripper can flexibly adapt to various shapes of objects.

## 2. Materials and Methods

### 2.1. Design of Granular-Tendon Robot Hand

As shown in Figure 1, the granular-tendon robot hand consisted of three main parts: two symmetrical fingers (left (L-) and right (R-)), a palm, and a driving actuator. Each finger comprised a rigid skeleton system and a soft part that mimicked a human finger bone and human finger skin, respectively. The skeleton system consisted of three links: distal phalanx, middle phalanx, and proximal phalanx, which were interconnected via the distal and middle joints. The distal phalanx included the fingertip with a shaft edge that imitated a human fingernail. The proximal phalanx was linked to the palm through a proximal joint at the palm adaptor. These hinge joints allowed two neighbouring phalanxes to only rotate about the centre shaft of the joint. Additionally, one end of the proximal phalanx was equipped with a roller structure for bearing the tendon.

A granular bag was designed to generate a soft structure for each phalanx. The surface of this bag was made of latex and had a block shape with a small thickness. Small particles were filled inside the rubber skin and attached to each phalanx. The fingers had four bags at the proximal and middle phalanxes of the same size and volume, while the volume of the bag at the distal phalanx was bigger than the others. Between wall-to-wall neighbouring granular bags there existed a vacant space to enhance the movement of each phalanx.

The palm comprises an L-shaped plate and a base plate. The L-shaped plate had two L-shaped brackets for holding a DC servo motor with the centre shaft parallel to the base plate. The motor is connected to the palm which houses the driving actuator system. The upper surface of the L-shaped plate contains spacers and bolts used to mount the palm onto the robot arm’s end effector. A driving pulley is fixed to the motor shaft for driving the robot fingers. To enhance the structural stiffness, the pulley is positioned over a long shaft with two fulcrums: one at the motor shaft and another at the motor-support block. The L-shaped plate is linked with the base plate using long spacers and bolts at the parallel and perpendicular surfaces, respectively. The base plate features palm adaptors for mounting the fingers.

There are a total of six tendons which drive the motions of the fingers, where two tendons, rl and rr, perform the releasing motion, and four tendons, gr and gl, generate the grasping motion. All tendons were made non-elongated during operation. Each grasping tendon is locked at the distal phalanx and passes through the holes in the middle and proximal phalanxes, palm adaptor, and palm before terminating at the driving pulley (see Figure 1). Each finger has one pair of grasping tendons with a symmetrical design. One end of a spring cord is fixed at the fingertip, while its remaining end locks the releasing tendon. This tendon lies on the roller in the proximal phalanx, reeves the palm adaptor and palm, and terminates at the driving pulley. According to the illustration in Figure 1c, six tendons are always straight from the pulley to the palm. In addition, the groups of grasping and releasing tendons are mounted on opposite sides of the pulley. When the driving pulley rotates in the clockwise direction, the grasping and releasing tendons are, respectively, pulled and released. In this scenario, the fingers bend towards each other to generate the grasping action. Conversely, the releasing action is implemented when the pulley rotates in the counterclockwise direction. During the entire operation, the total length of the tendons always change and the spring cord compensates for this variation.

**Figure 2:**
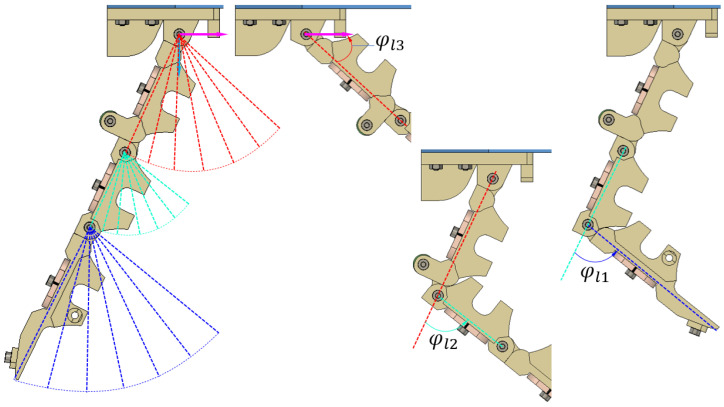
Schematic illustration of the skeletal motion without the granular bags. The red, green, and blue dashed lines indicate the position of each phalanx: proximal, middle, and distal phalanx, respectively, as they rotate around the hinge joints. In this figure, the red and green dashed lines are straight lines connecting the centres of two neighbour joints, whereas the blue dashed line starts from the centre of the distal joint to the fingertip. $\varphi_{l1},\varphi_{l2}$, and $\varphi_{l3}$ are, respectively, the rotational angles of each phalanx.

In Figure 2, the distal and middle phalanxes rotate corresponding to the angles $\varphi_{l1}$ and $\varphi_{l2}$ being 78°, whereas the proximal phalanx can move in the range of 72°. This is the upper limit of the finger skeleton in its freedom state. However, after adding the tendons and granular bags to each finger, the range of $\varphi_{l1},\varphi_{l2}$, and $\varphi_{l3}$ may be reduced. Moreover, increasing the space between two neighbour granular bags enlarges such angles.

### 2.2. Fabrication

**Figure 3:**
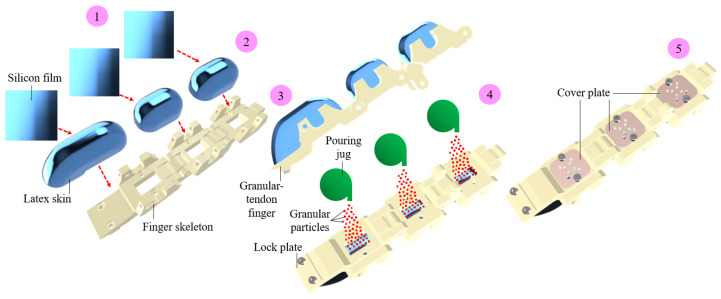
Step-by-step fabrication process for a granular-tendon finger. (1) Step 1: Prepare the latex skins. (2) Step 2: Generate the shape for the latex skin. (3) Step 3: Print the rigid skeleton and mount the granular bags over it. (4) Step 4: Pour the granular particles into the granular bags. (5) Step 5: Attach the cover plates to the phalanx for sealing the granular bags.

The granular-tendon finger designed in Figure 1 was fabricated using the process shown in Figure 3. The finger skeleton was 3D printed using PLA plastic on a Prusa 3D printer. Three latex sheets, each with a thickness of 1 mm were prepared and formed into capsule shapes. This step involved adding the latex sheet into a mould which generated the targeted shape of a granular bag. The latex capsules were subsequently fixed to the phalanx using glue films which generated soft skin for the finger. At this stage, each skin and its corresponding phalanx formed a vacant chamber at their inner surface. In the fourth step, granular particles were gradually poured into the vacant chamber through the groove on the phalanx. The particles were compressed and covered up using a cover plate. In this study, particles with a 2 mm diameter made from foam were used. The independent phalanx with the granular bag was linked together by the hinge joints to create the granular-tendon finger.

The driving pulley, L-shaped plate, base plate, palm adaptor, and pulley support were 3D printed using PLA plastic material on the Ender-3 3D printer. M3-steel spacers were mounted to form the frame structure of the palm. A DC servo motor (Coreless 35 kg) was attached to the palm at its shoulders. Six Kevlar tendons were used as grasping and releasing tendons. In this configuration, one end of each tendon was fastened to the driving pulley and secured with lock washers. The remaining ends of the grasping tendons were fixed on the distal phalanx, while those of the releasing tendon connected to a rubber band (8 × 1 mm${}^{2}$ cross-section) acted as the spring cord. The granular-tendon fingers were then fixed onto the palm adaptors and the driving pulley to the motor shaft. The tendon’s tension was adjusted to balance both the L- and R-finger. Finally, the pulley shaft and pulley support were assembled on the base plate to stabilize the driving pulley and DC motor.

## 3. Results

This section shows the experimental setup which determined the key parameters of the gripper in operation and validated the grasping performance of the granular-tendon gripper in picking various kinds of objects.

### 3.1. Finger Morphology

**Figure 4:**
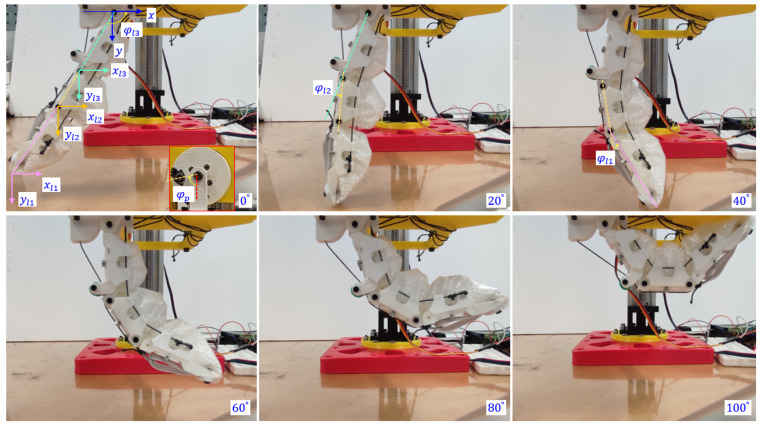
Evaluating the rotational angles $(\varphi_{l1},\varphi_{l2}$, and $\varphi_{l3})$ according to the variation in the rotational angle on the driving pulley $\varphi_{p}$. At each value of $\varphi_{p}$, the position of each joint and the fingertip in *x* and *y* directions were investigated.

**Figure 5:**
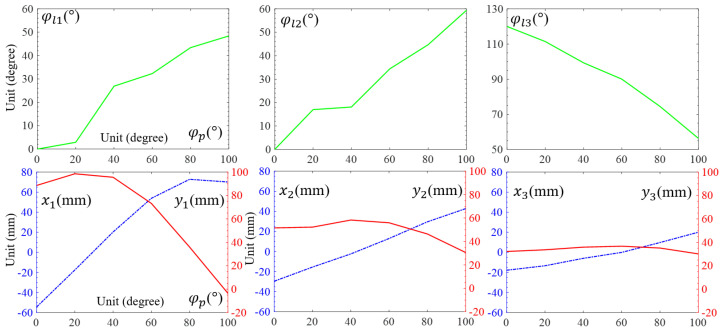
The position of each phalanx parameter $(\varphi_{l1},\varphi_{l2},$ and $\varphi_{l3})$ and ($x_{1},y_{1};x_{2},y_{2};x_{3},y_{3}$) according to the variation in $\varphi_{p}$. In each inset image, the blue dashed line and red solid line, respectively, show the data of x and y for each phalanx.

The morphology of the granular-tendon finger was determined using the coordinates $\left(x_{1},y_{1}\right),\left(x_{2},y_{2}\right)$, and $\left(x_{3},y_{3}\right)$ or, equivalently, the rotational angles $(\varphi_{l1},\varphi_{l2},$ and $\varphi_{l3})$ (see Figure 4). These values were influenced by the rotational angle of the pulley $\varphi_{p}$, which was set to zero when the finger was completely straight and in its maximum released state. Increasing $\varphi_{p}$ led to bending the finger inward. The values of x,y, and φ of the phalanx were obtained by image processing with their outcomes shown in Figure 5. $\varphi_{l1}$ and $\varphi_{l2}$ were zero at the maximum released state and reached 48.4° and 59.4° at $\varphi_{p}$ = 100°, whereas those of $\varphi_{l3}$ were, respectively, 120 and 56.3°. $\varphi_{l1}$ and $\varphi_{l2}$ increased, respectively, by 0.5 and 0.6°, and $\varphi_{l3}$ decreased by 0.6° for each degree deviation of $\varphi_{p}$. The reduction in $\varphi_{l3}$ followed a nearly linear trend, whereas the increments in $\varphi_{l1}$ and $\varphi_{l2}$ were not consistent. In particular, $\varphi_{l1}$ exhibited a rapid increment trend in the range of $\varphi_{p}$ = [20, 40]° while $\varphi_{l2}$ showed a similar trend in the range of $\varphi_{p}$ = [0, 20]°.

Coordinates x and y of each phalanx varied according to the variation in $\varphi_{p}$. As shown in Figure 5, $x_{1}$ increased from −54.4 to 72.8 mm and then slightly reduced to 70.1 mm in the range of $\varphi_{p}$ = [0, 80]° and [80, 100]°. Concurrently, $y_{1}$ reached the maximum value of 98.5 mm at $\varphi_{p}$ = 20°. $x_{2},x_{3}$ had a roughly linear increment with the (min, max) values, respectively, (−29.6, 42.7) and (−17.8, 20.1) at $\varphi_{p}$ = (0, 100)°. $y_{2}$ reached the maximum value 58.3 mm at $\varphi_{p}$ = 40°, whereas the maximum value of $y_{3}$ was 36.7 mm at $\varphi_{p}$ = 60°. The coordinate deviations in the phalanxes reduced from the distal to proximal; that of $x_{3},y_{3}$ was smaller compared to that of $x_{1},y_{1}$.

**Figure 6:**
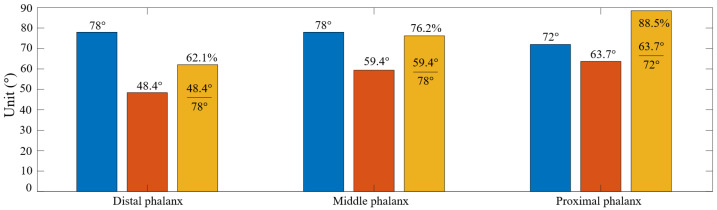
Influence of granular bag on the reduced rotational angle on each phalanx after assembly. At each group, the blue and yellow columns, respectively, illustrate the maximum angle of $(\varphi_{l1},\varphi_{l2}$, and $\varphi_{l3})$ before and after mounting the granular bags on the skeleton. The orange column shows the percentage ratio between the two previous columns at $\varphi_{p}=100^{{}^{\circ}}$.

As seen in Figure 6, the proximal phalanx achieved the highest deviation in the rotational angle with 63.7°. It reached 88.5% of the maximum value when there was no tendon and granular bag. Meanwhile, the middle and proximal phalanxes achieved 48.4° and 59.4°, corresponding with 62.1% and 76.2% of the maximum values. Thus, due to the constraints imposed by the tendons and granular bags, the finger could not reach the maximum rotational angle values of the skeleton shown in Figure 2. Furthermore, increasing $\varphi_{p}$ led to increased compression between the granular bags.

### 3.2. Finger Bearing Load

**Figure 7:**
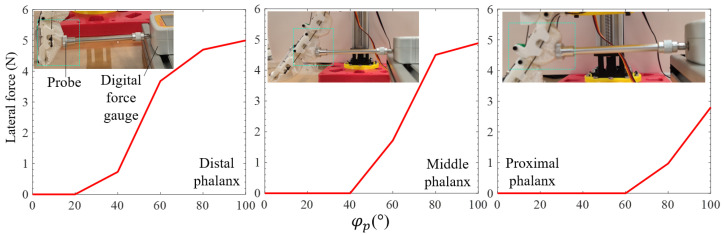
Testing the lateral force generated from the finger according to the variation in $\varphi_{p}$. The experiment was conducted for investigating the force at each phalanx. The green dashed line box indicates the testing location on the finger.

Figure 7 illustrates the lateral force generated by the finger during grasping. To obtain the force, the experiment was performed on the L-finger after the R-finger was removed. The setup included one digital force gauge with a range of 50 N fixed on the floor, such that the centre line of the main shaft was located within the finger plane and parallel to the floor. Moreover, one flat probe was attached to the furthest end on the left-hand side of the main shaft. Initially, the granular-tendon finger was completely straightened, and the gripper was positioned such that the apex of the granular bag (of the distal phalanx) slightly touched the probe. The motor drove the driving pulley in the counterclockwise direction and pulled the grasping tendons. The experiment was conducted at six levels of $\varphi_{p}$, 0, 20, 40, 60, 80, and 100°. At each trial, the digital force gauge indicated the lateral force generated by the finger at each phalanx. After the completion of the test on the distal phalanx, tests were performed to measure the lateral forces generated on the middle and proximal phalanxes.

The test outcomes revealed that there was hysteresis between $\varphi_{p}$ (the rotational angle of the driving pulley) and the lateral force exerted by the finger in the three phalanges. In the case of the distal phalanx, the lateral force remained constant when $\varphi_{p}$ increased to 20°, whereas, in the case of the middle and proximal phalanxes, the variation in lateral force appeared at $\varphi_{p}$ = 40° and 60°, respectively. The lateral force gradually increased after each 20° deviation except for a sudden jump between 40 and 60°. The middle phalanx exhibited a rapid increment in lateral force when $\varphi_{p}$ was between 40° and 80°, followed by a slight increase until maximum $\varphi_{p}$. The lateral force in the case of the proximal phalanx jumped from zero to 2.8 N in the range of $\varphi_{p}$ = [60, 100]°. The maximum lateral force measured in this design was generated by the distal phalanx with a value of 5 N, whereas, the middle phalanx created 97.8% lateral force generated by the distal case.

The lateral force obtained from the measurement depended on the displacement of the phalanx in the x-direction and the state of the phalanx (φ). According to Figure 5, we have the following sequence: $\Delta x_{1}>\Delta x_{2}>\Delta x_{3}$. This relation affected the maximum value of the lateral force observed in Figure 7. Due to the flexibility of granular particles, the probe created deformation on the soft skin of the finger at a small angle $\varphi_{p}$. As the granular bags became hard, the finger transferred the lateral force to the force gauge. In this gripper design, the lateral force was the primary component to hold and maintain a grip on the grasped objects. Consequently, the distal phalanx could achieve more stability in gripping compared to the middle and proximal phalanxes.

### 3.3. Grasping Performance

**Figure 8:**
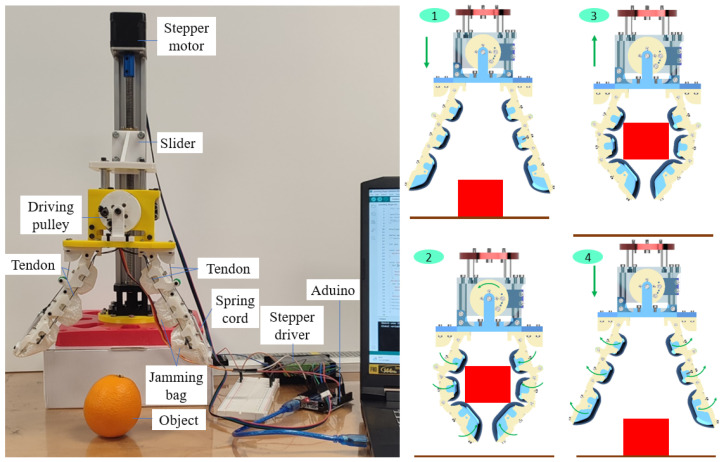
Experimental setup for evaluating the grasping performance of the granular-tendon gripper. The grasping process included four minor sequential steps: (1) approach, (2) grasp, (3) lift, and (4) release.

This section demonstrates the performance of the granular-tendon gripper in gripping various objects. The gripper was first mounted onto a linear actuator which provided the vertical motion for the gripper. The end effector of the hand was fixed to the slider of the linear actuator, and the translated motion of the slider was controlled with a Bachin (12V) stepper via a driving screw. The stepper motor was connected to a stepper driver and an Arduino Uno circuit which synchronously controlled the DC (6V) servo motor (see Figure 8).

Initially, the gripper was located such that the fingertips were positioned 10 mm above the floor and the fingers fully opened in the maximum state (with the straight phalanxes). The objects (15 different samples) were positioned on the floor: cylinder, U-shaped bracket, M3 bolt, tape, pyramid, big pyramid, orange, cake, coffee sachet, sphere, drink sachet, shelf, pulley gear, aluminium profile, and flat bracket (see Table 1). The fingers of the gripper then closed together to grasp the object on the floor. The gripper held and lifted the object to a height of 75 mm from the floor. Finally, the gripper moved back to its original position and released the object onto the floor. Each sample was tested five times before moving to the next sample.

**Figure 9:**
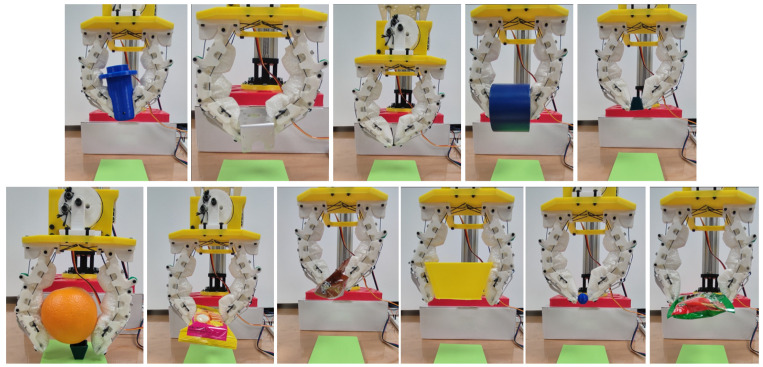
Demonstration of grasping various objects with the granular-tendon gripper involving successful cases. The objects shown in this figure (from left to right and top to bottom) were used: cylinder, U-shaped bracket, M3 bolt, tape, pyramid, orange, cake, coffee sachet, big pyramid, sphere, and drink sachet.

**Figure 10:**
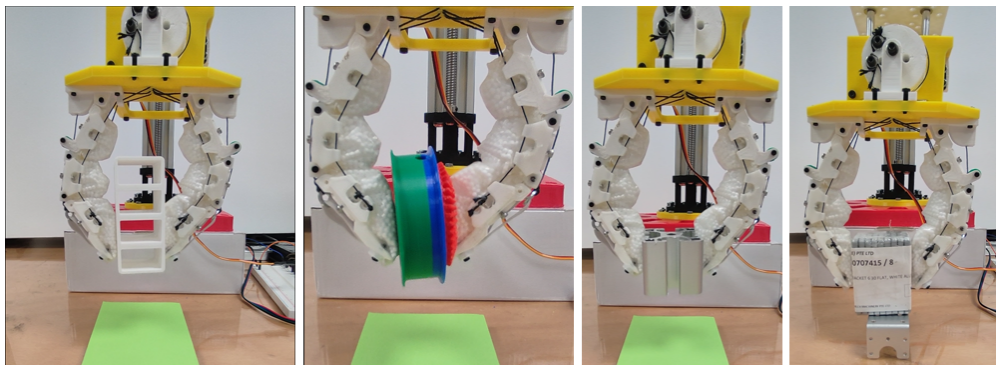
Demonstration of grasping various objects with the granular-tendon gripper without success cases. The objects in this figure (from left to right) were used: shelf, pulley gear, aluminium profile, and flat bracket.

Figure 9 demonstrates the grasping performance of the granular-tendon gripper with successful trials. The specific contact point between the gripper and the objects varied depending on their size. For light and small objects such as the bolt, pyramid, cake, coffee sachet, sphere, and drink sachet, the gripper used its fingertips to grip them. The granular bag of the distal phalanx was deformed to enhance the contact area with the objects, while the main grasping force was generated by the fingertips. The U-shaped bracket and tape were held by the distal phalanx with contact at that point. The cylinder and orange contacted with the fingers at both the distal phalanx and the middle phalanx on the L-finger. In this scenario, the grasping force was primarily exerted by the distal phalanx, while the middle phalanx enhanced the stability of the grasped objects. The big pyramid contacted both the middle phalanx and distal phalanx and received grasping force from both. As shown in Figure 10, the gripper could pick and lift all objects except for the flat plate. In this scenario, all objects contacted the finger at the distal phalanx with completely flat–flat contact interfaces.

**Figure 11:**
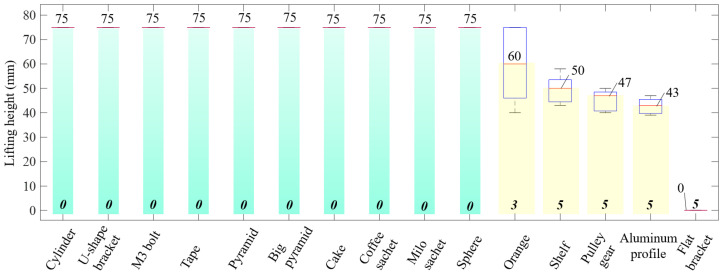
Evaluation of grasping objects by the lifting height and the number of failure cases. The red line indicates the mean value after five trials. Additionally, the displayed number at the bottom of each bar is the number of failure cases (between zero and five). The green and yellow columns, respectively, show the regions of no failure cases and failure cases.

Based on the results shown in Figure 11, when picking up the cylinder, U-shaped bracket, M3 bolt, tape, pyramid, big pyramid, cake, coffee sachet, drink sachet, and sphere, the gripper achieved a lifting height of 75 mm. Figure 11 also indicated the mean and deviation values of the grasped object samples that could not be lifted to the full height. In this situation, the gripper could not lift the flat bracket away from the floor. The mean value of all trials in this showcase was zero. The range of the lifting height for the case of grasping orange, shelf, pulley gear, and aluminium profile interfered. The mean values of lifting height for such cases were, respectively, 60, 50, 46.8, and 42.9 mm. While the mean value of grasping the orange was higher compared to the others, it also had the lowest minimum value and reached a maximum value of 75 mm. As shown in Figure 11, if one trial reached the full lifting height, we counted it as one successful case, and a failure case otherwise. In this scenario, grasping the shelf, pulley gear, aluminium profile, and flat bracket completely failed. Grasping the orange achieved two successful trials, whereas the remaining cases had zero failure cases.

As seen in Figure 9 and Figure 10, the granular particles facilitated the soft skins to adapt to various surfaces of the objects due to the morphological deformation. In fact, the objects with shaft edges such as the cylinder, U-shaped bracket, and the big pyramid generated more penetration than the remaining cases. The deformation on the skin was reduced when the gripper picked up the soft objects or other objects with flat contact interfaces. In fact, soft-body objects or objects that generated large deformation in the skin enhanced the success rates for grasping performance. Objects with heavy bodies caused instability when the gripper moved the large distance of the lifting height. Thus, the failure rate increased in the cases of grasping the shelf, pulley gear, aluminium profile, flat bracket, and orange. Although the tape weight was also heavy, the fingers could penetrate the vacant space inside it and scoop. In this scenario, the rigid skeleton driven by the tendons created a stable structure for bearing the load in the vertical direction.

## 4. Discussion

The hybrid design of the granular-tendon gripper demonstrates its capability to universally adapt to multiple objects of diverse sizes and weights, from light to heavy, which can be mounted to the robot arm [52], therapy robot hands [53], and soft haptic fingers [54]. The gripper exhibited high success rates in picking up small- and big-size objects. In this study, the granular bag was designed separately before being assembled into a finger. This enhanced the operation range of each phalanx and the finger during grasping. By utilizing the DC motor, the gripper was able to bear a heavy load in the vertical direction, while maintaining moderate lateral force. However, with two symmetrical fingers, the gripper had not generated enough energy to hold the objects with large inertia. Future investigations could explore the use of different granular particles, adjusting the volume of granular bags, and selecting stronger DC motors for picking up heavier loads. In this scenario, numerical estimations [55,56,57] are useful to investigate the deformation. The artificial skin [58,59,60] can be covered outside the robot hand for sensing and friendly interaction.

## 5. Conclusions

The experimental results confirmed that the granular-tendon finger could work with large angles of the phalanx. The gripper exhibited great adaptability to multiple shapes of objects owing to the soft structure of the latex skin and the granular particles. Additionally, the rigid structure of the skeleton and the tendon-driven mechanism enhanced the stiffness of the fingers during grasping. This study showed that the integration of multiple granular bags into the rigid structure of the finger skeleton enhanced the flexibility and stiffness of the gripper during grasping objects. This design approach inherited the advantages of both the soft and traditional grippers, offering improved adaptability compared to the jamming grippers with one granular bag. Future developments aim to enhance the stiffness of the gripper by considering the shape/size of the granular particles and the materials used for the latex skin.

## 6. Patents

The content of this study was filed a patent on 20 February 2023 and accorded PCT application number PCT/EP2023/054129.

## Figures and Tables

**Table 1:** Physical properties of the objects.

Object	Weight (g)	Maximum Dimension (mm)	Properties
Cylinder	23	72	rigid
U-shape bracket	13.3	66	rigid
M3 bolt	1.4	25	rigid
Tape	136.4	66	rigid
Pyramid	2.2	20	rigid
Big pyramid	20	85	rigid
Orange	210	55	soft
Cake	32.1	60	soft
Coffee sachet	31	180	soft
Sphere	1.5	15	rigid
Drink sachet	28.5	110.7	soft
Shelf	40	57	rigid
Pulley gear	30	65	rigid
Aluminum profile	64	30	rigid
Flat bracket	425.1	43	rigid

## Data Availability

Data sharing not applicable.
